# Laser Treatment Modalities for Diabetic Retinopathy

**DOI:** 10.7759/cureus.30024

**Published:** 2022-10-07

**Authors:** Gauri S Pande, Pravin Tidake

**Affiliations:** 1 Ophthalmology, Jawaharlal Nehru Medical College, Datta Meghe Institute of Medical Sciences, Wardha, IND

**Keywords:** neovascularization, retinal detachment, proliferative diabetic retinopathy, diabetic macular edema, argon, laser

## Abstract

Diabetes is a chronic progressive metabolic disorder that is caused by the body’s inability to regulate blood glucose levels. If uncontrolled, it can lead to various complications. Among its various complications, long-term diabetes leads to diabetic retinopathy (DR). It is a disease involving blood vessels and the destruction of retinal nerves. It is usually classified into two types: proliferative diabetic retinopathy (PDR) and nonproliferative diabetic retinopathy (NPDR). It progresses and causes loss of vision. The leading cause of loss of vision is diabetic macular edema (DME). The argon laser is used as a modality in the management of PDR. There are various types of laser photocoagulation, such as peripheral retinal laser photocoagulation, focal macular laser photocoagulation, and grid photocoagulation. DR results in various adverse consequences such as vitreous hemorrhage, fibrosis, traction, detachment of the retina, and glaucoma. To assess DR, a detailed fundus examination with a slit lamp biomicroscope needs to be done. Seven-standard field stereoscopic-color fundus photography needs to be done for documentation and follow-up. Patients with diabetes mellitus (DM) type 1 have a greater risk of suffering from DR. Another major complication of the condition is DME, which is characterized by an increase in the permeability of vessels and the thickening of the central part of the retina along with the accumulation of hard exudates on the macula. This article discusses various laser therapy modalities for the treatment of DR, their types, mechanisms, and aims. Clinical features of DR include abnormal dilatation of capillaries, and outpouchings in the form of microaneurysm from the capillary wall are one of the earliest and most dangerous changes; later, non-perfusion of the retina occurs, which is associated with cotton wool spots and blot hemorrhages. In patients suffering from PDR and maculopathy, peripheral retinal laser photocoagulation is used as a mode of intervention.

## Introduction and background

Diabetes mellitus (DM) is a progressive disease and, if longstanding, it will lead to cataracts and diabetic retinopathy (DR) [1]. DR, a complication of long-term uncontrolled DM, is a disease involving blood vessels. At any given time, patients with diabetes suffer from some kind of retinopathy. It is usually classified into two types: proliferative diabetic retinopathy (PDR) and nonproliferative diabetic retinopathy (NPDR). The basis of loss of sight is the formation of abnormal new vessels in the retina called neovascularization. It can cause various complications like vitreous hemorrhage, fibrosis, traction, and detachment of the retina. Clinically significant macular edema is also a complication of DR, which can occur at any stage of DR. It is mainly marked by an increase in vascular permeability, thickening of the central part of the retina called the macula, and the deposition of hard exudates on the macula. It is termed macular edema due to the swelling present on the macula. The leading reason for loss of sight in patients with DR is diabetic macular edema (DME) [2].

DM is an accelerating pathologic condition that results from the body's inability to regulate blood glucose levels [3]. DR is usually characterized by lesions involving the vessels and the destruction of the nerves of the retina [4]. The synthesis of new abnormal vessels in the retina is the cause of PDR. PDR can lead to vision loss. Argon lasers have been traditionally used as an intervention in PDR. Worldwide, there are roughly 93 million people with DR, of which 17 million have PDR, 21 million suffer from clinically significant macular edema, and 28 million suffer from DR with threatened sight loss [5]. In a time period of 25 years, nearly 42% of people with diabetes type 1 will develop PDR [6]. In an online survey, it was found that retinal photography was invariably selected as the basic method for screening the retina [7]. DR causes complications such as clinically significant macular edema, which causes impaired vision and, if not treated, can lead to vision loss. It will further deteriorate productivity and quality of life, which will result in an increase in socioeconomic burdens on society [8].

## Review

DR is a very serious complication of diabetes. It has become the primary reason for the loss of vision globally. Its overall occurrence is on the rise. Early detection and well-timed management of this vision-threatening DR will decrease the occurrence and development of sight loss. Screening for DR (using ophthalmoscopy and photography of the fundus) is precise, secure, and economical [9]. Examination of the fundus is relatively cheap and hence is performed in routine practice for screening the chronic or longstanding complications of diabetes. The fundus examination is economical and is used in routine practice for the screening of chronic diabetes complications. There is an increased association of cardiovascular mortality risk with DR in both diabetes type 1 and type 2. It is mostly associated with type 2 DM, whereas there are fewer studies on type 1 DM [10].

Seven-standard field stereoscopic-color fundus photography, which should be evaluated by a trained reader, is considered the standard screening modality for DR, though direct ophthalmoscopy or indirect slit-lamp fundoscopy through the dilated pupil or digital fundus photography may also be used [11]. At the later progressive stages of DR, PDR will cause more harm to vision and even lead to total loss of vision [12]. The development of DR depends not only on a person’s glycemic control but also on other factors such as genetic heritability. Heredity often determines a person's susceptibility to DR [13].

Characteristics of diabetic retinopathy

DR is characterized by retinal damage, specifically to retinal vessels, which in turn is caused due to uncontrolled blood sugar levels. Dilatation of capillaries is one of the earliest changes observed. The next thing to happen is the closing of capillaries, which causes a decrease in the supply of blood to some part of the retina, which is termed non-perfusion of the retina. On fundus examination, large pale areas are seen on the retina as they are devoid of blood supply. But in cases of smaller zones, they can be seen only by fluorescein angiography (FA). In this procedure, retinal blood vessels can be detected when the dye is passed through the vein and is then passed through the artery. The most crucial feature of DR is occluded capillaries, which lead to the stoppage of blood supply to the retina. The closing of capillaries is also associated with other two characteristic features. They are cotton wool spots. There is also the presence of dot and blot hemorrhages, which is due to hemorrhage of retinal infarcts. The appearance of greyish-white colored patches on the retina is called cotton wool spots. It appears due to infarcts on the retina. If many greyish-white patches are found (more than 6-10 in one eye), then it may be suggestive of rapid extensive retinal ischemia, but more commonly in DR, there is the presence of fewer cotton wool spots. And in the case of hemorrhages, they appear in various sizes and shapes often called "dot and blot". The smaller red-colored dots present on the retina are called micro-aneurysms, which result from the abnormal dilatation of capillaries. Differentiation of hemorrhages and micro-aneurysms cannot be done by performing ophthalmoscopy. Another cause of arterial damage is the thickening of the arterial wall and also the decrease in the width of the lumen known as stenosis, as well as arterial occlusion, which causes decreased blood supply to some parts of the retina [14]. There are biomarkers that are helpful in detecting the disease. DR-related biomarkers are found in blood, retina, vitreous, and aqueous humor and have also recently been found in tears [15]. The development of DR occurs progressively and pregnancy is a risk factor for it [16]. DR is graded according to the clinical features associated with it. Table 1 shows the grades of DR and associated clinical features.

**Table 1:** Classification used by the Scottish Diabetic Retinopathy Grading System* *[14] NPDR: nonproliferative diabetic retinopathy; ETDRS: Early Treatment for Diabetic Retinopathy Study

Grades	Clinical features
R0	No retinopathy
R1	Background: microaneurysms, retinal hemorrhages, with/without any exudate. This is broadly equivalent to the ETDRS mild NPDR stage
R2	Pre-proliferative: multiple blot hemorrhages, intraretinal microvascular abnormalities (IRMAs). Moderate NPDR, referable to ophthalmology
R3	Proliferative diabetic retinopathy

Pathophysiology of diabetic retinopathy

Histopathological findings associated with DR include the loss of pericytes and cells of the endothelium, as well as basilar membrane thickening. Biochemically, there will be a synthesis of advanced glycation end products, caused by the rise in the activity of protein kinase C (PKC) and glycosylation of proteins. All these processes will cause communication between the cells, which also include vascular endothelial growth factor (VEGF), which functionally causes the production of new vessels in both the segments of the eye - anterior as well as posterior - as well as an increase in the permeability of the retinal vessels, and distortion of the barrier between blood and retina [17].

Pathophysiology of NPDR

It is characterized by abnormal permeability of the capillaries of the retina, which leads to retinal edema, and also the closing of capillaries, which leads to non-perfusion of the retina and ischemia.

Pathophysiology of PDR

When retinal ischemia is severe enough to lead to neovascularization, it is called PDR. It will further progress to vitreous detachment and vitreous hemorrhage, which leads to vision loss. If not treated appropriately, 50% of patients suffering from PDR will lose their vision in the span of five years [6].

Mechanisms of diabetic retinopathy

It involves several pathways such as oxidative stress, an increase in pro-inflammatory mediators, and an increase in the secretion of VEGF secretion. These factors will cause the development of micro-aneurysms, non-perfusion of capillaries, leakage from vessels, and the formation of new vessels, which is termed neovascularization [18]. Oxygen metabolism of the retina plays a very crucial role in a variety of diseases. Retinal hypoxia is generally a result of an underlying disease and it will cause the generation of VEGF. Hypoxia will lead to ischemia and extensive ischemia will lead to neovascularization of the retina. Macular edema is a result of an increase in vascular permeability and a rise in capillary hydrostatic pressure. On the other hand, new vessels are developed on the retina as a result of the physiological attempt of the eye to balance the hypoxia, and this will result in neovascularization of the retina. But the newly formed vessels are weak and hence may cause vitreous hemorrhage. And especially in the case of diabetes mellitus, it can also be associated with fibrosis, which will in turn increase the risk of traction retinal detachment [19]. Several studies have reported that anti-VEGF agents, if added to surgical interventions, will decrease the rate of formation of new vessels also known as neovascularization, and will also cause a decrease in bleeding and further complications, and for vitreous hemorrhage, it should be followed by endo-laser [20].

Assessment of diabetic retinopathy

The American Diabetes Association (ADA) has suggested that patients suffering from type 1 DM should examine the retina within five years of getting diagnosed with diabetes. They should also undergo regular life-long retinal examinations [21]. In diabetic patients without clinically observable DR, optical coherence tomography angiography (OCTA) is used to interpret vessels of the retina and capillaries parameters [22]. Systems including photographs use readers in the form of human or artificial intelligence for the detection of the disease. Readers are used for analyzing the pictures of the fundus and identifying structural features of DR and also seek predictions about the risk of loss of sight based on its severity [23]. It has been observed that there is a reduction in the number of patients with vision loss pertaining to DR due to improved and timely examination of people with diabetes along with an improvement in glycemic control [24].

Types of laser treatment modalities

Peripheral Retinal Laser Photocoagulation

In the case of patients suffering from pre-proliferative retinopathy, this intervention will lower the occurrence of severe visual loss (SVL) [1]. It is usually carried out in areas of hypoxia, areas that are supplied with less oxygen in the periphery of the retina [25]. People with proliferative retinopathy benefit more from laser photocoagulation than those with proliferative maculopathy.

Focal Macular Laser Photocoagulation

It is mainly performed in patients with focal DME plus mild to moderate pre-proliferative DR as it will decrease the probability of moderate visual loss.

Grid Photocoagulation

In cases of diffuse maculopathy, grid photocoagulation can be done in areas of the retina, which has been thickened to enhance vision. In general, photocoagulation is not likely to be effective in eyes with maculopathy but without DME. Maculopathy is a disease of the macula and DME occurs when blood vessels leak into a part of the retina called the macula [2]. According to the Early Treatment of Diabetic Retinopathy Study (ETDRS), the benchmark for the treatment of PDR is argon laser photocoagulation [5]. A newer type of laser photocoagulation has been developed, which is known as subthreshold, in which laser burns of lesser energy are given and hence they will cause less damage [26].

Features of laser photocoagulation

Laser photocoagulation is a treatment modality very commonly used to manage DR and prevent further complications. In this intervention, light energy is applied to achieve the goal of ceasing the production of new blood vessels, which stops the neovascularization and in turn will prevent vision loss. It can be given in one sitting or, in order to reduce the chances of side effects, in multiple sittings. New treatments are on the rise, and hence the use of laser therapy may become less prevalent in higher-income countries but will still be relevant as it is economical. New modalities such as anti-VEGF agents may lead to a decline in the use of laser photocoagulation in developed and high-income countries, but it will still remain relevant because it is suitable in other parts of the world [25]. The addition of steroids reduces inflammation, which is caused due to laser, and hence, in turn, laser power can be decreased [27]. Earlier, laser photocoagulation was the basic treatment modality for PDR but it is being progressively replaced in the management of DME. In the case of patients with PDR, if laser treatment is provided in a timely manner, vision loss can be prevented; however, it is not very effective in reversing already decreased vision [12].

Mechanism of laser photocoagulation

Laser photocoagulation decreases the oxygen demand of the retina, which will help to redirect enough oxygen and nutrients to the retina, thereby changing the hemodynamics. Secondly, it also acts by reducing the occurrence of vasoactive factors such as VEGF and PKC [4]. Direct laser burns are applied to neovascular tufts to coagulate the vessels, which ceases neovascularization. For feeder vessel condensation, both xenon arc and argon laser photocoagulation are used. But in the current scenario, physicians use argon more widely than xenon since xenon causes more complications than argon [28]. Among the VEGF family, VEGF-A is currently believed to be most involved in the pathogenesis of a variety of situations related to the retina because of its role in the production of new vessels. Permeability of vessels results in the destruction of barriers between blood and retina [29]. A small quantity of laser burns is applied to single vessels. The heat of the light is used in focal laser photocoagulation to mask or destroy the atypical retinal vessels [25].

Aim of laser photocoagulation

VEGF is produced in the areas of ischemia, and when the laser is applied to non-ischemic areas, it coagulates the tissues, which decreases the oxygen demand of the peripheral retina, which will stop the production of VEGF. After the energy is absorbed by the pigments of the tissues, thermal damage will occur and the laser acts by this way [5].

Adverse effects of laser photocoagulation

Laser is associated with various side effects. These reportedly include foveal burns, visual field defects, retinal fibrosis, and scars due to lasers. Also, there may be sub-retinal and vitreous bleeding, and it may also lead to epiretinal proliferation with retinal traction. It can affect the ability of a person to drive a vehicle. Therefore, laser therapy is given only after all these factors are carefully studied and considered [14]. While laser photocoagulation is very useful, it also causes visual complications comprising choroidal effusions, retinal detachment with exudates, DME, defects of the visual field, and defects in vision in dim light. The above-mentioned complications occur due to various effects of lasers such as an increase in exposure timing, power, and giving all of the treatment in a single sitting, and all these will cause increased distribution of heat energy in the choroid and the retina [30]. Also, there are some rare complications of retinal laser photocoagulation, which include weakness in the accommodation of the eye, dilatation of the pupil with myopia, which is transient in nature, and sensitivity of the cornea being impaired badly [31].

Diabetic Macular Edema

ETDRS has mentioned primary DME as it results from the disruption of the barrier between blood and retina, which is secondary to leakage occurring from micro-aneurysms, capillaries, and arterioles from the retina. Factors contributing to the increased occurrence of DME include hypertension, cardiovascular disease, abnormal functioning of the renal system, and poor control of blood glucose [32]. Fluid accumulation at the macula causes a decrease in visual acuity, which may be reversible if short-lived, but if prolonged edema is present, irreversible damage can occur, which can result in permanent vision loss. The most common presenting symptom of DME is the blurring of vision [33]. The complications of DR [34] are presented in Table 2.

**Table 2:** Incidence of complications in a given population* *[34]

Complications	Number of studies	The proportion of patients with events at each time point	Total number of patients at risk at each given time
Incidences	-	-	-
Proliferative diabetic retinopathy	12	15.6	7,204
Severe vision loss	6	7.4	9,468
Diabetic macular edema	5	12.0	2,430
Photocoagulation	6	7.8	5,948
Retinopathy >2 step	3	45.1	2,342

Studies have shown that DME patients who were treated with laser photocoagulation had fewer chances of developing vision loss in comparison with the patients who were not given laser in a span of three years [26]. After 1985, there has been a decline in cases of PDR and SVL progressing due to diabetes. In the last three decades, there has been a decrease in the rate of DR as suggested by some studies, which can be attributed to the improvement in awareness among both patients and clinicians, the increase in screening for the disease, as well as the improvement in the proper treatment and management of DM [34]. In patients with DR, the most common reason for vision loss is DME, with an increased incidence in people with type 2 DM [35].

## Conclusions

In this article, we have examined various laser treatment modalities for DR. Also, we have discussed in detail the pathology of DR, its clinical features, clinical assessment, and related complications. Prolonged DM leads to several complications, one of which is DR. To assess DR patients, a detailed fundus examination is done. Clinical features of DR include dilatation of capillaries, which is also one of the earliest changes, and non-perfusion of the retina, which is associated with the presence of cotton wool spots. Dot and blot hemorrhages are also seen due to hemorrhage of retinal infarcts. Laser photocoagulation has been the most commonly used intervention for DR for a long period of time; however, newer interventions such as intravitreal anti-VEGF are used more frequently nowadays. Argon laser is commonly preferred over xenon, as xenon laser causes more complications. Thermal energy is used to cease the production of new retinal vessels, which is also called neovascularization. Secondly, it also acts by decreasing the synthesis of vasoactive peptides, which are VEGF and PKC. Feeding vessels are coagulated by applying direct heavy burns, which stops neovascularization. There are various adverse effects of laser photocoagulation, and these include foveal burns, visual field defects, retinal fibrosis, and scars due to lasers. It can also affect the ability of a person to drive. Laser photocoagulation is very useful, but as with any other surgery, it also causes certain complications such as choroidal effusions, exudative retinal detachment, DME, defects of visual fields, and defects in vision at night. Additionally, there are some rare complications of retinal laser photocoagulation, which include weakness in the accommodation of the eye, dilatation of the pupil with myopia, which is transient in nature, and sensitivity of the cornea being impaired badly. Despite all these drawbacks, laser photocoagulation is still the most efficient treatment available to prevent vision loss in patients with DR.
